# Insulin Withdrawal in Diabetic Kidney Disease: What Are We Waiting for?

**DOI:** 10.3390/ijerph18105388

**Published:** 2021-05-18

**Authors:** Carlos Morillas, Luis D’Marco, María Jesús Puchades, Eva Solá-Izquierdo, Carmen Gorriz-Zambrano, Valmore Bermúdez, José Luis Gorriz

**Affiliations:** 1 Endocrinology Department, Hospital Doctor Peset, 46020 Valencia, Spain; cmorillas@telefonica.net (C.M.); eva.sola@uv.es (E.S.-I.); 2 Nephrology Department, Hospital Clínico Universitario, INCLIVA, Universidad de Valencia, 46010 Valencia, Spain; luisgerardodg@hotmail.com (L.D.); chuspuchades@gmail.com (M.J.P.); 3 CAP Sant Pere, ABS Reus 1, 43202 Tarragona, Spain; carmengorrizz@gmail.com; 4 Facultad de Ciencias de la Salud, Universidad Simon Bolivar, Barranquilla 080001, Colombia; valmore@gmail.com

**Keywords:** diabetic kidney disease, cardiovascular disease, GLP-1RA, SGLT2i, insulin

## Abstract

The prevalence of type 2 diabetes mellitus worldwide stands at nearly 9.3% and it is estimated that 20–40% of these patients will develop diabetic kidney disease (DKD). DKD is the leading cause of chronic kidney disease (CKD), and these patients often present high morbidity and mortality rates, particularly in those patients with poorly controlled risk factors. Furthermore, many are overweight or obese, due primarily to insulin compensation resulting from insulin resistance. In the last decade, treatment with sodium–glucose cotransporter 2 inhibitors (SGLT2i) and glucagon-like peptide-1 receptor agonists (GLP1-RA) have been shown to be beneficial in renal and cardiovascular targets; however, in patients with CKD, the previous guidelines recommended the use of drugs such as repaglinide or dipeptidyl peptidase-4 inhibitors (DPP-4 inhibitors), plus insulin therapy. However, new guidelines have paved the way for new treatments, such as SGLT2i or GLP1-RA in patients with CKD. Currently, the new evidence supports the use of GLP1-RA in patients with an estimated glomerular filtration rate (eGFR) of up to 15 mL/min/1.73 m^2^ and an SGLT2i should be started with an eGFR > 60 mL/min/1.73 m^2^. Regarding those patients in advanced stages of CKD, the usual approach is to switch to insulin. Thus, the add-on of GLP1-RA and/or SGLT2i to insulin therapy can reduce the dose of insulin, or even allow for its withdrawal, as well as achieve a good glycaemic control with no weight gain and reduced risk of hypoglycaemia, with the added advantage of cardiorenal benefits.

## 1. Introduction

The prevalence of type 2 diabetes mellitus (T2DM) worldwide stands at nearly 9.3% (463 million people) [1], and it is estimated that 20–40% of these patients will develop diabetic kidney disease (DKD) 10–15 years after diagnosis [2]. DKD is the leading cause of chronic kidney disease (CKD) globally, and these patients often present considerable morbidity and mortality rates, particularly in the case of patients with poorly controlled blood pressure and metabolic disorders, such as hyperglycaemia and dyslipidaemia [3]. Furthermore, many are overweight or obese, due primarily to insulin compensation resulting from insulin resistance. The NAHNES III study (2007–2012) analyzed 1380 patients with T2DM and revealed that 17% of the patients with CKD were receiving treatment with insulin (16.2%, 27.5%, 24.9%, 22.9% and 38% in stage 1, 2, 3, 4 and 5 of CKD, respectively), and 64% of them were obese (body mass index [BMI] >30 kg/m^2^) [4]. In the last decade, treatment with sodium–glucose cotransporter 2 inhibitors (SGLT2i) and glucagon-like peptide-1 receptor agonists (GLP1-RA) have been shown to be beneficial in renal and cardiovascular (CV) targets; however, in patients with CKD, the previous guidelines [5] recommended the use of repaglinide or dipeptidyl peptidase-4 inhibitors (DPP-4 inhibitors), together with insulin therapy, with basal insulin (BI) at onset and progressing later to basal-bolus (BB) therapy. Very recently, new guidelines have paved the way for new treatments, such as SGLT2i or GLP1-RA in patients with CKD, but with some limitations. Currently, the new evidence supports the use of GLP1-RA (liraglutide, semaglutide or dulaglutide) in patients with an estimated glomerular filtration rate (eGFR) of up to 15 mL/min/1.73 m^2^ (in Spain, if BMI 30 kg/m^2^) [6,7,8]. Despite the evidence, treatments with SGLT2i should be started with an eGFR > 60 mL/min/1.73 m^2^. To date, only canagliflozin can be used in stage 3 CKD. Given the evidence, this point in particular needs to be updated. However, no guidelines recommend or have issued statements regarding insulin treatment in those patients with advanced stages of CKD, in whom the introduction of these new drugs may include CV benefits [5,9]. Regarding those patients in advanced stages of CKD, the usual approach is to switch to insulin due to the limitations on metformin and sulfonylurea use in patients with a reduced eGFR. Despite its cardiovascular (CV) and renal benefits, GLP1-RA is not widely used in many countries, and in some cases barely reaches 10% [10].

## 2. What We Know: Advantages of New Therapeutic Agents (SGLT2i and GLP1-RA)

At present, we are acutely aware of the deleterious effects of hyperinsulinism (endogenous or exogenous in diabetic patients to whom insulin is administered) with its consequent weight-gain and sodium reabsorption in the proximal tubule which increases the risk and incidence of heart failure [11]. Insulin resistance and hyperinsulinism alter the systemic and neurohumoral environment, causing changes in metabolism, changes in insulin, signaling in cardiomyocytes developing in the impaired heart, and adverse left ventricular remodeling, contributing to myocardial dysfunction [12]. On the other hand, hyperinsulinism is associated with renal structure damage, mainly in renal tubules and interstitial cells of blood vessels [13].

**Figure 1:**
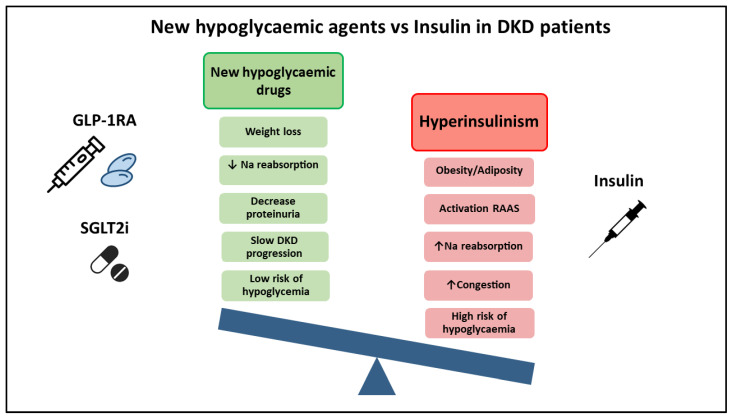
SGLT2i and GLP-1RA could be used to avoid the hyperinsulinism effects. The treatment with insulin is associated to weight gain, RAAS activation, increased sodium reabsorption in the renal proximal tubule, congestion and increased risk of hypoglycaemia. On the contrary, the new therapeutic options have proved renal benefits, such as proteinuria decrease, slowing renal progression, and less sodium reabsorption. Moreover, they have a weight loss effect with less risk of hypoglycaemia. Abbreviations: Sodium–glucose cotransporter 2 inhibitors, SGLT2i; glucagon-like peptide-1 receptor agonists, GLP1-RA; estimated glomerular filtration rate, eGFR; Sodium, Na; Renin—angiotensin—aldosterone system, RAAS.

Several consensus documents from different scientific societies currently recommend the prescription of GLP1-RA and/or SGLT2i with proven CV and renal benefits in patients at high risk or with DKD, regardless of their HbA1c levels. However, these guidelines do not indicate how to reduce or stop insulin dosing in those patients under treatment [5,9]. Meanwhile, many of these patients with DKD and obesity receiving insulin therapy could benefit from reduced insulin doses with the introduction of these novel cardio and nephroprotective drugs. As a result, weight gain, hypoglycaemia risk factors, and water retention could be avoided (Figure 1).

## 3. What We don’t Know

The de-escalation of insulin therapy in overweight and insulin-resistant patients may be beneficial, since data from recent, real-life studies suggests that insulin can be reduced or suspended in those patients under treatment, though not all studies achieved this reduction in the dose of insulin therapy [14,15,16]. Traditionally, patients with uncontrolled T2DM had insulin treatment optimized from BI therapy to multiple doses of insulin (MDI) with BB therapy, not achieving optimal glycaemic control and increasing the risk of hypoglycaemia, with the deleterious effects of water retention and an increase in the body weight.

The new treatments for T2DM, mainly SGLT2i and GLP1-RA, have changed the CV and renal scenario, as they are able to achieve stricter control objectives with less adverse outcomes, such as hypoglycaemia, while improving weight control and reducing the need for insulin. However, although less in proportion, they also have undesirable side effects to consider (Table 1).

There is evidence to support the use of C-peptide as a reliable parameter for the evaluation of pancreatic reserve [17] and the calculation of the residual secretory capacity of pancreatic β-cells in subjects with T2DM (insulinized or non-insulinized), that could help in the withdrawal of insulin if the insulin reserve is adequate [18]. Monoclonal antibody assays, such as chemiluminescence or fluorescence, have made the determination of C-peptide more sensitive and specific, improving its reproducibility. At present, the interference with proinsulin is less than 10%. However, caution should be exercised in interpreting the results of different studies [19] because analytical standardization has not been fully achieved. C-peptide can be measured during fasting, but in patients treated with insulin, pancreatic secretion may be suppressed, so the post-stimulus determination (intravenous glucagon or mixed-meal) is considered more useful.

## 4. What Do the Guidelines Say? Why Don’t the Guidelines Recommend Insulin Withdrawal?

The guidelines suggest prescribing insulin in the progression of a staggered treatment but make no statement on how to reduce or withdraw insulin in those insulinized patients, primarily obese, who should be included in these new therapeutic regimens with renal and CV benefits, regardless of the degree of metabolic control.

These guidelines tend to be gradual, depending on the deterioration of metabolic control, and indicate how to start insulin therapies, how to increase the dose of insulin, or when to add rapid insulin BBs, but there are no suggestions on how to decrease or withdraw insulin once the therapy is started (when no up-to-date evidence was available on the benefits and safety in DKD with SGLT2i and GLP1-RA). The characteristics of SGLT2i, and especially GLP1-RA, due to its hypoglycaemic potency, may permit a de-escalation of insulin, or even a withdrawal, in the case of a preserved insulin reserve and if only a few years have passed since the time of a diabetes diagnosis. 

The AWARD 7 study shows that adding on weekly doses of a GLP1-RA (dulaglutide) to the administration of insulin glargine yielded results similar in metabolic control, but superior in renal benefits in patients with DKD using pre-prandial rapid insulin BB [18]. Moreover, in all studies in which a GLP1-RA was added, the BI doses were reduced. As an example, in the SUSTAIN 5 study with semaglutide, the BI dose was reduced by 20% before adding GLP1-RA [20]. Thus, by adding semaglutide to insulin therapy, the insulin dose was reduced, primarily in those patients with a baseline HbA1c under 8.0%.

Recently, the SPARE study [21], a real-life study carried out in endocrinology clinics in Canada that included patients who started GLP1-RA with their usual treatment, reported that adults using BB therapy required a significantly lower median total daily dose of insulin following the add-on of weekly doses of semaglutide (0.82 vs. 0.93 U/kg; *p* < 0.001).

## 5. Suggestions Considering the Current Evidence

The current evidence regarding the renal and CV benefits of these new therapeutic groups (SGLT2i, and especially GLP1-RA) and the possibility of their administration to insulinized patients with DKD, paves the way for new scenarios in which insulin doses can be reduced, or even withdrawn in patients with obesity, or in those with adequate insulin reserve (measured by baseline C-peptide or after intravenous glucagon stimulation).

With the introduction of a GLP1-RA in patients with a BB regimen, rapid insulin boluses could be avoided. Consequently, we would be able to focus on fasting glycemia control to maintain baseline blood glucose levels between 80 and 130 mg/dL with a BI dose adjustment every 3 days. In the case of patients with mixed insulin regimens, 50–60% of the total dose could be switched to BI with an add-on of weekly GLP1-RA therapy, making the same adjustments to achieve baseline fasting glucose ranges between 80 and 130 mg/dL. Thus, in patients with only one dose of BI, the introduction of SGLT2i, or especially a GLP1-RA, could reduce the dose of insulin by 20% in cases of acceptable metabolic control (HbA1c 8%), and the patients could rely on self-control to achieve fasting glycemia between the suggested ranges.

If the BI requirement after adjustments is 0.2 U/kg/day or 15 U/day, insulin withdrawal could be evaluated, and SGLT2i and/or GLP1-RA could be maintained or added. Moreover, even though switching patients from a BB insulin regimen to a GLP1-RA added on to BI appears to be a beneficial option for patients who have not received a GLP1-RA in the past, it would seem more reasonable to initiate a GLP1-RA even before adding prandial insulin to BI, especially in those patients with cardiac or renal comorbidities [22].

Very recently Naing et al. published a 16-week randomized clinical trial in obese patients with T2DM and advanced insulin therapy [23]. The patients were randomized to intervention (step-down) the insulin plus adding SGLT2i and GLP1 RA (all with metformin) with discontinuation of all prandial insulin injections vs. a control group that remined with MDI injections, as previously. De-escalating from advanced insulin therapy to the combined use of metformin, SGLT2i, GLP1-RA and BI in obese patients with poorly controlled T2DM on MDI resulted in significant improvements in glycaemic control, weight loss and significantly higher patient satisfaction. This stepping-down approach may be the better option than continuing MDI in these patients and support our hypothesis.

## 6. Conclusions

In summary, the add-on of GLP1-RA and/or SGLT2i to insulin therapy can reduce the dose of insulin, or even allow for its withdrawal (in the case of preserved insulin reserve, primarily in patients diagnosed with diabetes in recent years), as well as achieve an ideal triad in the treatment (good glycaemic control with no weight gain and reduced risk of hypoglycaemia), with the added advantage of cardiorenal benefits. Therefore, this therapeutic strategy could improve the management of patients with T2DM and DKD with obesity or preserved insulin reserve, as it can be safely administered in those patients with impaired kidney function (eGFR > 15 mL/min/1.73 m^2^).

## Figures and Tables

**Table 1:** Comparison of adverse or undesirable effects of insulin and other drugs uses in DKD patients.

Adverse Effects	Insulin	SGLT2i	GLP1-RA
Hypoglycaemic risk	+++	+	+
Weight gain	+++	-	-
GI discomfort	-	+	+++
Ketoacidosis	++	+++	+
Volume depletion	-	++	+
Orthostatic hypotension	-	++	+
Genital infection	-	++	-

Abbreviations: Sodium-glucose cotransporter 2 inhibitors, SGLT2i; glucagon-like peptide-1 receptor agonists, GLP1-RA; Gastrointestinal; GI.

## Data Availability

Not applicable.

## References

[1] Saeedi P., Petersohn I., Salpea P., Malanda B., Karuranga S., Unwin N., Colagiuri S., Guariguata L., Motala A.A., Ogurtsova K. (2019). Global and regional diabetes prevalence estimates for 2019 and projections for 2030 and 2045: Results from the International Diabetes Federation Diabetes Atlas, 9th edition. Diabetes Res. Clin. Pract..

[2] Alicic R.Z., Rooney M.T., Tuttle K. (2017). Diabetic Kidney Disease. Clin. J. Am. Soc. Nephrol..

[3] Chen H.-Y., Sun C.-Y., Lee C.-C., Wu I.-W., Chen Y.-C., Lin Y.-H., Fang W.-C., Pan H.-C. (2021). Ketoanalogue supplements reduce mortality in patients with pre-dialysis advanced diabetic kidney disease: A nationwide population-based study. Clin. Nutr..

[4] Wu B., Bell K., Stanford A., Kern D.M., Tunceli O., Vupputuri S., Kalsekar I., Willey V. (2016). Understanding CKD among patients with T2DM: Prevalence, temporal trends, and treatment patterns—NHANES 2007–2012. BMJ Open Diabetes Res. Care.

[5] de Boer I.H., Caramori M.L., Chan J.C., Heerspink H.J., Hurst C., Khunti K., Liew A., Michos E.D., Navaneethan S.D., Olowu W.A. (2020). KDIGO 2020 Clinical Practice Guideline for Diabetes Management in Chronic Kidney Disease. Kidney Int..

[6] Marbury T.C., Flint A., Jacobsen J.B., Karsbøl J.D., Lasseter K. (2017). Pharmacokinetics and Tolerability of a Single Dose of Semaglutide, a Human Glucagon-Like Peptide-1 Analog, in Subjects With and Without Renal Impairment. Clin. Pharmacokinet..

[7] Jacobsen L.V., Flint A., Olsen A.K., Ingwersen S.H. (2016). Liraglutide in Type 2 Diabetes Mellitus: Clinical Pharmacokinetics and Pharmacodynamics. Clin. Pharmacokinet..

[8] Jacobsen L.V., Hindsberger C., Robson R., Zdravkovic M. (2009). Effect of renal impairment on the pharmacokinetics of the GLP-1 analogue liraglutide. Br. J. Clin. Pharmacol..

[9] Cosentino F., Grant P.J., Aboyans V., Bailey C.J., Ceriello A., Delgado V., Federici M., Filippatos G., Grobbee D.E., Hansen T.B. (2020). 2019 ESC Guidelines on diabetes, pre-diabetes, and cardiovascular diseases developed in collaboration with the EASD. Eur. Heart J..

[10] Arganda C. IQVIA Estima el Impacto de la OPR en Oficina de Farmacia. Diario Farma 2020. https://www.diariofarma.com/2020/12/03/iqvia-estima-el-impacto-de-la-opr-en-oficina-de-farmacia-en-34-millones-y-deja-al-mercado-sin-crecimiento.

[11] Cas A.D., Khan S.S., Butler J., Mentz R.J., Bonow R.O., Avogaro A., Tschoepe D., Doehner W., Greene S.J., Senni M. (2015). Impact of Diabetes on Epidemiology, Treatment, and Outcomes of Patients With Heart Failure. JACC: Hear. Fail..

[12] Riehle C., Abel E.D. (2016). Insulin Signaling and Heart Failure. Circ. Res..

[13] Zhang Y., Yang S., Cui X., Yang J., Zheng M., Jia J., Han F., Yang X., Wang J., Guo Z. (2019). Hyperinsulinemia Can Cause Kidney Disease in the IGT Stage of OLETF Rats via the INS/IRS-1/PI3-K/Akt Signaling Pathway. J. Diabetes Res..

[14] Naing S., Ramesh G., Garcha J., Poliyedath A., Khandelwal S., Mills P. (2020). SUN-LB115 Is the Stepping-Down Approach a Better Option Than Multiple Daily Injections in Patients With Chronic Poorly-Controlled Diabetes on Advanced Insulin Therapy?. J. Endocr. Soc..

[15] Tofé S., Argüelles I., Mena E., Serra G., Codina M., Urgeles J.R., García H., Pereg V. (2018). Real-world GLP-1 RA therapy in type 2 diabetes: A long-term effectiveness observational study. Endocrinol. Diabetes Metab..

[16] Rentsch T., Awad M., Moorman J.M., Gothard M.D. (2020). Evaluating the Impact of Glucagon-Like Peptide-1 Receptor Agonists on Metabolic Changes in Patients With Type 2 Diabetes on High-Dose Insulin. Am. J. Ther..

[17] Goto A., Takaichi M., Kishimoto M., Takahashi Y., Kajio H., Shimbo T., Noda M. (2010). Body Mass Index, Fasting Plasma Glucose Levels, and C-peptide Levels as Predictors of the Future Insulin Use in Japanese Type 2 Diabetic Patients. Endocr. J..

[18] Tuttle K.R., Lakshmanan M.C., Rayner B., Busch R.S., Zimmermann A.G., Woodward D.B., Botros F.T. (2018). Dulaglutide versus insulin glargine in patients with type 2 diabetes and moderate-to-severe chronic kidney disease (AWARD-7): A multicentre, open-label, randomised trial. Lancet Diabetes Endocrinol..

[19] Jones A.G., Hattersley A.T. (2013). The clinical utility of C-peptide measurement in the care of patients with diabetes. Diabet. Med..

[20] Rodbard H.W., Lingvay I., Reed J., De La Rosa R., Rose L., Sugimoto D., Araki E., Chu P.-L., Wijayasinghe N., Norwood P. (2018). Semaglutide Added to Basal Insulin in Type 2 Diabetes (SUSTAIN 5): A Randomized, Controlled Trial. J. Clin. Endocrinol. Metab..

[21] E Brown R., Bech P.G., Aronson R. (2020). Semaglutide once weekly in people with type 2 diabetes: Real-world analysis of the Canadian LMC diabetes registry ( SPARE study). Diabetes Obes. Metab..

[22] Bolli G.B., Porcellati F., Meier J.J. (2020). Switching From Insulin Bolus Treatment to GLP-1 RAs Added to Continued Basal Insulin in People With Type 2 Diabetes on Basal-Bolus Insulin. Diabetes Care.

[23] Naing S., Ramesh G., Garcha J., Poliyedath A., Khandelwal S., Mills P.K. (2021). Is the stepping-down approach a better option than multiple daily injections in obese patients with poorly controlled Type 2 diabetes on advanced insulin therapy?. Endocrinol. Diabetes Metab..

